# Functional polymorphism in aldehyde dehydrogenase-2 gene associated with risk of tuberculosis

**DOI:** 10.1186/1471-2350-15-40

**Published:** 2014-04-02

**Authors:** Seung Kyu Park, Choon-Sik Park, Hyo-Suk Lee, Kyong Soo Park, Byung Lae Park, Hyun Sub Cheong, Hyoung Doo Shin

**Affiliations:** 1 Sorokdo National Hospital, 65 Doyang-eup, Goheung-gun, Jeonnam 548-904, Republic of Korea; 2 Genome Research Center for Allergy and Respiratory Diseases, Soonchunhyang University Bucheon Hospital and Seoul Hospital, Seoul, Republic of Korea; 3 Department of Internal Medicine and Liver Research Institute, Seoul National University, 28 Yungun-dong, Chongro-Gu, Seoul 110-744, Republic of Korea; 4 Department of Internal Medicine, Seoul National University College of Medicine, Seoul 110-744, Republic of Korea; 5 Department of Genetic Epidemiology, SNP Genetics, Inc., 1 Shinsu-dong, Mapo-gu, Seoul 121-742, Republic of Korea; 6 Department of Life Science, Sogang University, 1 Shinsu-dong, Mapo-gu, Seoul 121-742, Republic of Korea

**Keywords:** Aldehyde dehydrogenase, Tuberculosis, Polymorphism

## Abstract

**Background:**

The well-known genetic polymorphisms in *ADH1B(His47Arg)* and *ALDH2(Glu487Lys)* have dramatic effects on the rate of metabolizing alcohol and acetaldehyde. We investigated possible involvement of these functional polymorphisms in other common complex-trait diseases.

**Methods:**

The genetic effects of these two polymorphisms on hepatitis, asthma, type-2 diabetes mellitus (T2DM), and tuberculosis (TB) were examined in a Korean population.

**Results:**

We demonstrated that the well-known functional polymorphism of a primary alcohol-metabolizing enzyme *(ALDH2 Glu487Lys)* has a strong genetic association with the risk of TB. The frequency of the minor allele (ALDH2*487Lys) was found to be much lower in TB patients (freq. = 0.099/n = 477) than among controls (freq. = 0.162/n = 796) (P = 0.00001, OR (95% confidential interval) = 0.57 (0.45-0.74)). Our data may indicate that TB was once an endemic disease, which exerted selection pressure for higher frequencies of ALDH2*487Lys in Asian populations. In addition, the calculated attributable fraction (AF) indicates that 39.5% of TB patients can attribute their disease to the detrimental effects of *ALDH2Glu487Glu.*

**Conclusion:**

Our results suggest that this polymorphism is one of the genetic components of TB, at least in the Korean population.

## Background

The alcohol dehydrogenases (ADHs), especially ADH1B, convert alcohol to acetaldehyde. Subsequently, the aldehyde dehydrogenases (ALDHs), mainly ALDH2, oxidate the acetaldehyde into acetate. Encoding genes for these two representative alcohol-metabolizing enzymes display polymorphisms (*ADH1B* His47Arg and *ALDH2* Glu487Lys) that show different alcohol/acetaldehyde oxidizing capability among individuals [1-4]. The ADH1B*47His allele represents a much higher activity of ADH1B with about 40 times higher Vmax than the homozygotes for the ADH1B*47Arg form, which enables increased alcohol elimination from the blood after alcohol consumption [1,5]. The ALDH2*487Lys allele encodes a catalytically inactive subunit [1,5], which causes alcohol-related adverse reactions [6]. These adverse reactions in subjects with ALDH2*487Lys, as a result of excessive acetaldehyde accumulation, tend to reduce alcohol consumption, consequently reducing the risk of alcoholism.

We previously demonstrated that ADH1B*47Arg and ALDH2*487Lys were dramatically associated with the risk of alcoholism in a Korean population [7,8]. In addition, when combined, the two loci revealed major genetic effects on the risk of alcoholism [9]. To investigate possible involvement of these functional polymorphisms in other common complex-trait diseases, we examined their genetic effects on HBV (hepatitis B virus) hepatitis, asthma, type-2 diabetes mellitus (T2DM), and tuberculosis (TB) in a Korean population.

## Methods

### Study subjects and genotyping of polymorphisms

Information about the study subjects was provided in previous studies (number of samples may differ slightly) as follows: HBV (hepatitis B virus) hepatitis [10], asthma [11], T2DM [12], and tuberculosis (TB) [13]. Briefly, the following subjects were included: a total of 963 subjects for the hepatitis study (537 chronic carriers [mean age = 55.0, range = 23-82] and 426 spontaneously recovered subjects from hepatitis B [mean age = 55.0, range = 29-79]), 1,796 subjects for the asthma study (1,259 asthma patients [mean age = 33.8, range = 22-65] and 537 controls [mean age = 37.1, range = 32-56]), 1,359 subjects for the T2DM study (745 T2DM patients [mean age = 57.1, range = 43-72] and 614 controls [mean age = 65.5, range = 45-76]), 1,032 subjects for the alcoholism study (549 alcoholics [all males; mean age = 46.1, range = 20–73] and 483 controls [all male; mean age = 33.2, range = 20–77]), and 1,273 subjects for the TB study (447 TB patients [all male; mean age = 46.7, range = 20–86] and 796 controls [all male; mean age = 54.9, range = 40–69]). Inclusion criteria were described in previous studies [11-13].

### Statistics

χ^2^ tests were used to determine if the individual variants were in Hardy-Weinberg equilibrium (HWE). Logistic regression analyses, controlling for age and gender as covariates, were used to calculate odds ratios and the *P*-values for case–control analysis. All statistical analyses were performed using SAS 9.1 (SAS Institute, Inc., Cary, NC, USA). The attributable fraction (AF) was calculated by the formula AF = *f*(*R* ⋅ - 1)/(1 + *f*(*R* ⋅ - 1)), where *f* is the frequency of the risk factor in the population, and *R* is the measure of the odds ratio [14].

## Results

The genotype distributions of *ADH1B* His47Arg and *ALDH2Glu487Lys* were not found to deviate from HWE (P > 0.05) except in alcoholic patients (*ADH1B* His47Arg). (For comparison, the results of our previous study of two polymorphisms and their association with alcoholism [9] have been added to Table 1). Logistic regression analysis did not show any significant association with the clearance of hepatitis B virus or with the risks of asthma, T2DM, or TB (Table 1). However, a strong genetic association of *ALDH2Glu487Lys* with the risk of TB was observed. The frequency of the minor allele was much lower in TB patients (freq. = 0.099/n = 477) than in controls (freq. = 0.162/n = 796) (P = 0.00001, OR (95% confidential interval) = 0.57 (0.45-0.74), Table 1). The overall allele frequencies of ALDH2*487Lys ranged from 0.161–0.181, except in alcoholic patients (freq. = 0.017) and TB patients (freq. = 0.099), in the Korean population.

**Table 1:** **Analysis of genetic effect of *ADH1B His47Arg *** **and *ALDH2 Glu487Lys *** **on the risk of various diseases in a Korean population (n = 6,423)**

**Loci**	**Disease**	**Group**	**Genotype**			**Total N**	**MAF**^**a**^	**HWE**^**b**^	**OR (95% CI)**^**c**^	**P**^**c**^
			**His/His**	**His/Arg**	**Arg/Arg**					
*ADH1B His47Arg*	Hepatitis	CC^d^	317(59.0%)	196(36.5%)	24(4.5%)	537	0.227	0.361	0.95 (0.76-1.18)	0.64
		SR^d^	243(57.1%)	165(38.7%)	18(4.2%)	426	0.236	0.125		
	Asthma	Case	708(56.2%)	464(36.9%)	87(6.9%)	1259	0.253	0.358	0.99 (0.84-1.17)	0.91
		Control	295(54.8%)	210(39.2%)	32(6.0%)	537	0.256	0.503		
	T2DM	Case	449(60.3%)	251(33.7%)	45(6.0%)	745	0.229	0.214	0.91 (0.76-1.08)	0.28
		Control	349(56.8%)	227(37.0%)	38(6.2%)	614	0.247	0.893		
	Alcoholism^e^	Case	217(39.5%)	145(26.4%)	187(34.1%)	549	0.473	**3.172 × 10**^**-28**^	**2.38 (2.00-2.82)**	**1.28E-22**
		Control	298(61.7%)	155(32.1%)	30(6.2%)	483	0.223	0.893		
	TB	Case	273(57.2%)	169(35.4%)	35(7.4%)	477	0.251	0.217	1.01 (0.84-1.22)	0.89
		Control	450(56.5%)	297(37.3%)	49(6.2%)	796	0.248	0.999		
*ALDH2Glu487Lys*	Hepatitis	CC	372(69.3%)	157(29.2%)	8(1.5%)	537	0.161	0.058	1.02 ( 0.80-1.31)	0.88
		SR	305(71.6%)	107(25.1%)	14(3.3%)	426	0.158	0.230		
	Asthma	Case	860(68.3%)	358(28.4%)	41(3.3%)	1259	0.175	0.617	1.17 (0.96-1.41)	0.12
		Control	385(71.7%)	139(25.9%)	13(2.4%)	537	0.154	0.914		
	T2DM	Case	498(66.8%)	224(30.1%)	23(3.1%)	745	0.181	0.718	1.05 (0.87-1.28)	0.60
		Control	426(69.4%)	163(26.5%)	25(4.1%)	614	0.173	0.066		
	Alcoholism^e^	Case	530(96.5%)	19(3.5%)	0(0.0%)	549	0.017	0.680	**0.10 (0.06-0.16)**	**3.88E-20**
		Control	346(73.6%)	122(25.3%)	15(3.1%)	483	0.265	0.297		
	TB	Case	391(82%)	78(16.4%)	8(1.7%)	477	0.099	0.082	**0.57 (0.45-0.74)**	**0.00001**
		Control	559(70.2%)	216(27.1%)	21(2.6%)	796	0.162	0.980		

^a^MAF: minor allele frequency.

^b^*P-*value of genotype distribution deviation from Hardy-Weinberg equilibrium.

^c^Logistic regression models (co-dominant model) were used for calculating odds ratios (95% confidential interval) and corresponding *P-*values.

^d^CC: chronic carrier/SR: spontaneously recovered.

^e^Kim et al. (2008) [9].

Bold faces indicate *P*<0.05.

Further referent and alternative (co-dominant, dominant, and recessive model) analysis of *ALDH2Glu487Lys* revealed that its genetic mode for the risk of TB is apparently protective and dominant, when comparing the magnitude (OR) of associations of heterozygotes and homozygotes for ALDH2*487Lys, although no significance was detected for homozygotes due to a low number of samples (Table 2). Only 18.1% of TB patients had the protective allele (ALDH2*487Lys), compared with 29.7% of controls (dominant mode; OR = 0.52 [95% confidential interval (CI), 0.39-0.69], P = 000004) (Table 2).

**Table 2:** **Analysis of genetic effect of *ALDH2 Glu487Lys *** **on risk of TB (n = 1,273)**

**Genotype**	**TB**	**Control**	**Referent analysis**^**a**^		**Co-dominant model**		**Dominant model**		**Recessive model**	
			**OR (95% CI)**	**P**	**OR (95% CI)**	***P***	**OR (95% CI)**	***P***	**OR (95% CI)**	***P***
Glu/Glu	391(82%)	559(70.2%)	1	-						
Glu/Lys	78(16.4%)	216(27.1%)	**0.52 (0.39-0.69)**	**0.000008**	**0.57 (0.45-0.74)**	**0.00001**	**0.52 (0.39-0.69)**	**0.000004**	0.63 (0.28-1.43)	0.27
Lys/Lys	8(1.7%)	21(2.6%)	0.55 (0.24-1.24)	0.15						

^a^Odds ratio and *P-*value of individual genotype as compared to common genotype in referent analysis.

Bold faces indicate *P*<0.05.

The AF was calculated by frequency of risk genotype (ALDH2*Glu/Glu, 70.2%) and its odds ratio (1.93) based on controls (Table 2). The AF indicates that 39.5% of TB patients can attribute their disease to the detrimental effects of ALDH2*Glu/Glu in the Korean population*.*

## Discussion

Tuberculosis is one of the oldest diseases known to mankind; it is also a leading cause of death among adults, and it kills almost 500 children each day worldwide. More than 90% of TB-related deaths occur in developing countries. The incidence of TB was declared a global emergency by the World Health Organization (WHO) in 1993, with around 8–10 million new cases of the disease each year and 2–3 million deaths worldwide (WHO Report, 2005). A worldwide health problem that reached a peak in the nineteenth century, it was thought to have been brought under control by the l960s due to active public health measures and the use of modern drug therapies. However, this complacency led to reduced funding for the diagnosis and treatment of TB at the same time that the bacterium was developing resistance to the drugs used to treat it. The problem has been compounded since the 1980s by the emergence of a new population of vulnerable individuals—those infected with HIV.

Approximately one third of the world’s population is latently infected with *Mycobacterium tuberculosis,* which causes TB. However, only 10% of those infected are estimated to progress to active (clinical) TB. A large body of epidemiological data links polymorphisms in various host genes to increased susceptibility to TB [15-17]. These host genetic factors are among the important determinants of susceptibility to the disease [18]. The doubly high risk of disease in identical twins compared with non-identical twins [19] strongly indicates a host genetic component. There have been many studies on TB susceptibility based on candidate-gene approaches. Recently, several genome-wide association studies (GWASs) identified susceptibility loci for tuberculosis on chromosome 18q11.2 [20], chromosome 11p13 [21], chromosome 20q12 [22], and eight genes involved in immune signaling [23].

In this study, we demonstrated that one functional polymorphism in a primary alcohol-metabolizing enzyme *(ALDH2Glu487Lys)* is a genetic component underlying susceptibility to TB in a Korean population. The protective effect of ALDH2*487Lys on TB could be explained through the well-known function of this variant in alcohol metabolism. Although ADH1B*47 is *involved* in alcohol elimination from the blood after consumption (alcohol to acetaldehyde), it is *not directly involved* in alcohol-related adverse reactions. However, the ALDH2*487Lys allele encodes an inactive converting enzyme (acetaldehyde into acetate), resulting in excessive accumulated acetaldehyde, which causes alcohol-related adverse reactions including flushing, palpitation, nausea, headache, drowsiness, breathlessness, and general discomfort [1,5,6]. These adverse reactions likely lead to reduced alcohol abuse, which in turn helps to maintain better general health and reduces the risk of TB, along with other confounding factors.

Interestingly, the genotype distribution of *ALDH2* differs greatly between Asian, especially East Asian, and other major populations. In fact, ALDH2*487Lys has only been detected in East Asian populations [24]. The hypothesis of selection pressure for lower ALDH2 (mediated by ALDH2*487Lys), against some endemic disease(s) in areas with high frequency of ALDH2*487Lys, seems like one of the most plausible explanations. It is not easy to prove, historically, given the time period during which it operated. However, based on the higher prevalence of TB and higher frequencies of ALDH2*487Lys in East Asian populations, a likely hypothesis is that TB is or was an endemic disease in Korean and possibly Japanese and Chinese populations. This could have exerted selection pressure that accounts for the higher frequencies of ALDH2*487Lys in Asian populations.

## Conclusions

In this paper, we demonstrated that the well-known functional polymorphism of a primary alcohol-metabolizing enzyme *(ALDH2 Glu487Lys)* has a genetic association with the risk of TB. We also offer evidence that TB may have been an endemic disease in Asian populations, which exerted selection pressure resulting in higher frequencies of ALDH2*487Lys. In addition, the AF indicates that alcoholism in 39.5% of TB patients in the Korean population can be attributed to the detrimental effect of ALDH2*487Glu, which suggests that this polymorphism is one of the genetic components of TB, at least among Koreans.

## Abbreviations

ADH: Alcohol dehydrogenase; ALDH: Aldehyde dehydrogenase; TB: Tuberculosis; AF: Attributable fraction.

## Competing interests

The authors declare that they have no competing interest to report.

## Authors’ contributions

SK Park and HS Cheong carried out the molecular genetic studies, participated in the sequence alignment, and drafted the manuscript. SK Park, HS Lee, and KS Park recruited samples in this study. BL Park participated in the genotyping. SK Park and HD Shin developed the design of the study and performed the statistical analysis. HD Shin conceived of the study, participated in its coordination, and helped to draft the manuscript. All authors read and approved the final manuscript.

## Pre-publication history

The pre-publication history for this paper can be accessed here:

http://www.biomedcentral.com/1471-2350/15/40/prepub
